# The Effects of Fungal Feed Additives in Animals: A Review

**DOI:** 10.3390/ani10050805

**Published:** 2020-05-06

**Authors:** Wen Yang Chuang, Yun Chen Hsieh, Tzu-Tai Lee

**Affiliations:** 1 Department of Animal Science, National Chung Hsing University, Taichung 402, Taiwan; xssaazxssaaz@yahoo.com.tw (W.Y.C.); richard840909@gmail.com (Y.C.H.); 2 The iEGG and Animal Biotechnology Center, National Chung Hsing University, Taichung 402, Taiwan

**Keywords:** fungi, probiotic, feed additive, mushroom waste compost

## Abstract

**Simple Summary:**

Fungal probiotics and ferments have potential as feed additives, but their use has long been ignored. The main goal of this review article is to report on the potential benefits and hazards of fungal feed additives. Previous research indicates that fungal feed additives enhance antioxidant capacity and decrease the inflammatory response in animals through polysaccharides, triterpenes, polyphenols, ergosterol, and adenosine. Accordingly, fungal feed additives could further enhance growth performance and animal health and could be of functional use.

**Abstract:**

As probiotics, fungi enhance animal health and are suitable animal feed additives. In addition to brewing fungi, there are also edible and medicinal fungi. Common fungi utilized in feeding programs include *Saccharomyces cerevisiae, Aspergillus oryzae*, *Pleurotus* spp., *Antrodia cinnamomea*, and *Cordyceps militaris*. These fungi are rich in glucans, polysaccharides, polyphenols, triterpenes, ergosterol, adenosine, and laccases. These functional components play important roles in antioxidant, anti-inflammatory, anti-obesity, and immune system regulation. As such, fungal feed additives could be of potential use when breeding livestock. In previous studies, fungal feed additives enhanced body weight and egg production in poultry and improved the feed conversion rate. Several mycotoxins can be produced by hazardous fungi but fortunately, the cell walls constituents and enzymes of fungal probiotics can also act to decrease the toxicity of mycotoxins. Overall, fungal feed additives are of value, but their safety and usage must be studied further, including cost-benefit economic analyses.

## 1. Introduction

Fungi, as ancient eukaryotes, have been present on Earth for at least 2.4 billion years [1], and include 120,000 different species [2]. Fungi have traditionally been classified according to their morphology but can now be identified by DNA. The existence of fungi is inseparable from human history and development. Fungi can be classified as either edible or fermenting, based on their usage. The former includes common fungi such as *Pleurotus eryngii*, *Flammulina velutipes*, *P. ostreatus*, and mushrooms; the latter includes probiotics such as *Aspergillus oryzae*. Currently, although studies have shown that *Pennisetum* can be used as a culture medium for edible fungi, most are still planted in space packages filled with wood chips [3,4]. Mature fungi are rich in minerals, vitamins, and probiotics and, due to their powerful antioxidative functions, are increasingly attractive to consumers [5]. However, due to the widespread cultivation of mushrooms, increasing attention has been paid to the treatment of mushroom waste compost, the residual culture medium [4,6].

*Saccharomyces cerevisiae* and *A. oryzae* have been integrated into human life since ancient times. The former is related mainly to alcohol production, while the latter is involved in the fermentation process of soy sauce. Chuang et al. [7] and Khempaka et al. [8] both pointed out that, following fermentation by *A. oryzae* or *Saccharomyces cerevisiae*, neutral detergent fibers decrease and hemicellulose and crude protein content increase in the fermented product. In addition, our research shows that *S. cerevisiae* and *A. oryzae* can be used as feed additives and assist in reducing the inflammatory response in animals [7].

In addition to probiotics and edible mushrooms, the earliest discovered antibiotic—penicillin—is also produced by fungi (*Penicillium*). With the discovery and use of antibiotics, animals can grow rapidly and be free of many diseases. However, the abuse of antibiotics in recent years has caused many microorganisms to develop resistance, therefore reducing antibiotics in the diet has become increasingly important. Since 2006, the European Union has banned antibiotic use in animal feed (Commission Implementing Regulation (EU) 1831/2003 and 1463/2004, European Parliament) and other countries are also heading in this direction. In order to reduce the use of antibiotics, beneficial fungi whose ferments do not produce antibiotics or mycotoxins have been investigated, due to their increased antioxidant capacity [9].

Mycotoxins are fungal toxins often found in improperly preserved feed whose negative effects on animals include decreased growth performance and intestinal and liver damage [10,11]. However, other fungal probiotics could reduce damage by encasing mycotoxins in their cell walls or even degrading them [7,12,13].

Although most of the discussion on probiotics has focused on bacteria, fungi have considerable potential as probiotics and have been ignored for too long. Over the past five years, our research team has investigated the effects of fungal feed supplements on poultry health [4,7,9,14,15,16,17,18,19,20,21]. Although there have been many studies on the use of fungi and their ferments as feed supplements and their effect on animal health, most review articles have focused on the addition of probiotics or the effects of mycotoxins. This review aims instead to explore the use of fungal feed additives and their potential risks.

## 2. Edible Fungi and Their Potential Uses

There are more than 2000 known species of edible fungi worldwide, several of which have been commercially cultivated, including medicinal fungi. Common medicinal fungi include *Cordyceps* spp. and *Antrodia* spp., while edible fungi include *Pleurotus* spp., *Lentinula* spp., *Agaricus* spp., and *Flammulina* spp. *Cordyceps militaris*, *Pleurotus eryngii*, *Pleurotus sajor-caju*, and *Flammulina velutipes* are 4 fungi renowned for their nutritional benefits to animals.

*Pleurotus eryngii*, *Pleurotus sajor-caju* and *Flammulina velutipes* are the most commonly cultured mushrooms in Taiwan (Taiwan Agricultural Research Institute, Council of Agriculture, Executive Yuan). According to the estimates of the Taiwan Agricultural Research Institute, the annual production volume of fresh mushrooms is 140,000 tons, and the output value exceeds 10 billion new Taiwan dollars. It is therefore important to exploit any added value of mushroom usage. Although wild *Cordyceps sinensis* has many functional components and strong biological activity, it takes a long time to grow under strict conditions in order to form fruiting bodies and its yield is insufficient for human needs. Furthermore, *Cordyceps sinensis* cannot be cultivated artificially and can only be harvested once. In contrast, *Cordyceps militaris* only takes one to three months to mature and therefore has greater potential for development and use. In addition, *Cordyceps militaris* can be cultivated in media and has functional components similar to *Cordyceps sinensis*, garnering it greater attention [22]. *Cordyceps militaris* is rich in a variety of bioactive compounds, such as cordycepin, polysaccharides, ergosterol, and mannitol [23]. Some studies have also extracted polysaccharides with antioxidative or immunomodulatory activities from *Cordyceps militaris* [24,25,26].

*Antrodia cinnamomea,* an endemic species in Taiwan, has been used for centuries due to its high antioxidative and anti-inflammatory capacities [27,28]. The major functional components in *A. cinnamomea* are polysaccharides, triterpenes, sterols, benzenoids, benzoquinone derivatives, and maleic acid. [29]. These components could enhance antioxidative capacities and reduce the damage from tumors [30]. According to Lee et al. [20], the addition of 0.1%, 0.2%, or 0.4% *A. cinnamomea* powder not only decreased the coliform count and increased lactobacilli in broiler intestines, but it also enhanced the antioxidative capacity in serum. As a result, *A. cinnamomea* was also considered as a potential feed additive in chicken.

*Pleurotus eryngii* contains many functional ingredients, such as polysaccharides and peptides, is high in fiber, and has high anti-inflammatory and antioxidative capacities [31]. Widely used by humans as an important edible mushroom [32], the whole plant can be processed and is used widely as a health food [32]. *Pleurotus sajor-caju*, a mushroom similar to *Pleurotus eryngii*, is low in calories with very low lipid and starch levels but is rich in protein, fiber, minerals, and vitamins [5]. *Pleurotus sajor-caju* has a variety of biologically active compounds, including polysaccharides, phenols, terpenes, and sterols. [5]. It also has antiviral, antibacterial, antifungal, antioxidative, and anti-inflammatory activities, and is therefore widely used in traditional medicine and nutritional research [33].

In addition to its delightful aroma and taste, *Flammulina velutipes* has a variety of pharmacological properties [34]. It is also well known for its curative properties in liver disease and gastroenteric ulcers [35]. Yang et al. [36] indicated that the extractable polysaccharides in *Flammulina velutipes* have high antioxidative capacities, while the mushroom itself has anti-cancer, antibacterial, and immunomodulatory properties [36]. Based on these characteristics, *Flammulina velutipes* is very promising for further research. However, no matter the species, one must keep in mind that when the mature mushroom is removed, the waste compost remains, potentially causing new environmental problems [4]. Previous research shows that mushroom byproducts have a similar nutritional composition as edible parts [37,38]. As such, based on its low cost, rich nutritional composition, large supply, and numerous biologically active functions, it has potential as a feed additive.

## 3. Hazardous Fungi Species

Mycotoxins are secondary metabolites produced by some pathogenic fungi. Mycotoxins can affect animal health and are one of the main detriments to growth [10]. Unfortunately, corn and soybeans, the main ingredients in animal feed, are prone to mold when improperly stored and may accumulate mycotoxins [11]. Common mycotoxins include aflatoxins, citrinin, ochratoxin, T-2 toxin, and vomitoxin [39]. Mycotoxins reduce growth performance in animals and are also nephrotoxic, hepatotoxic, and neurotoxic [10,40]. At present, the harm from mycotoxins can be reduced by mold adsorbents, mycotoxin-degrading enzymes, and probiotic degradation [12,40]. Interestingly, although produced by pathogenic fungi, mycotoxins can be treated with yeast cell walls or enzymes produced by edible fungi [12,40]. The main components in yeast cell walls, glucan, and mannan, are mycotoxin-adsorbing substances, while laccase and oxidase produced by *Pleurotus eryngii* reduce the toxicity of mycotoxins [12,13]. The main concern regarding the use of mushroom waste compost is whether its culture medium is conducive to mold growth and could produce mycotoxins. Fortunately, according to Loi et al. [12] and Haque et al. [13], the mushroom mycelium and enzymes contained in waste compost are not conducive to mycotoxin production. In addition, under proper storage conditions (including rapid drying and crushing), the risk of mycotoxins in mushroom waste compost can be further minimized.

## 4. The Functional Components of Fungi

**Figure 1:**
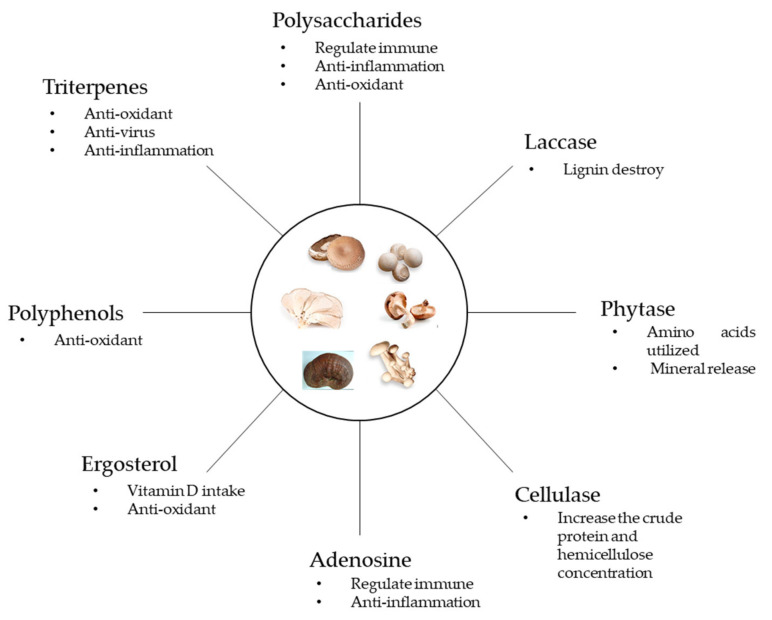
Fungal compounds and their potential functions. Mushroom images were adapted from Du et al. [42], and the photograph of *Ganoderma* was taken by the author.

During fungus cultivation, several enzymes assist the fungi in degrading and utilizing medium nutrients [7]. During this process, many fungal metabolites, secondary metabolites, and synthetics are produced [5,41] (Figure 1). These substances include fungal cell walls, polysaccharides, ergosterol, adenosine, and triterpenes. [5]. In addition to anti-inflammatory, antimicrobial, anti-fatigue, and anti-malarial effects, these functional substances can also protect the lungs and liver and enhance immunity and antioxidant capacity [41].

### 4.1. Triterpenes

Triterpenes are functional sterol metabolites commonly found in mushrooms, that are mainly present in lanostane [5,43]. However, although Chuang et al. [4] reported that triterpenes can be found in mushroom waste compost, most research has focused on triterpenes in medicinal fungi. Mushroom triterpenes can inhibit α-glucosidase activity [44] and thereby reduce blood sugar concentration in animals [45]. Triterpenoids present in *Ganoderma lucidum* can reduce LPS/ D-Galactosamine-induced liver damage by reducing tumor necrosis factor-alpha (TNF-α) and interleukin-6 (IL-6) expression [46]. These triterpenes can also inhibit TLR4-MyD88 (Toll-like receptor 4-myeloid differentiation primary response 88) activity and therefore decrease NF-κB expression [46,47]. In addition, Choi et al. [48] indicated that the anostane triterpene content in *G. lucidum* enhanced heme oxygenase-1 expression and decreased the inflammatory response in RAW264.7 cells. Novak [49] reported on the antibacterial activity of diterpenes in *P. mutilis*, while Ma et al. [43] described the functionality of other terpenoids in mushrooms, including antioxidant and anti-virus capacities.

### 4.2. Polyphenols and Flavonoids

Polyphenols are functional substances commonly found in nature, especially plants and fungi [50,51], with flavonoids being among the most effective [50]. Gil-Ramirez et al. [52] indicated there were no flavonoids in mushrooms, due to the absence of flavonoid absorption and synthesis enzymes. This claim was challenged four years later by Mohanta [53], who pointed to evidence that a large number of fungi contain genes and enzymes related to flavonoid synthesis.

According to Gil-Ramirez et al. [52], it comes down to four points: (1) the traditional method of measuring flavonoid content via absorbance is wrong; (2) flavonoids in the medium cannot be absorbed by the mushrooms; (3) mushrooms do not have genes or enzymes related to flavonoid synthesis; and (4) something went wrong in about 9% of the reports that used HPLC analysis to determine flavonoid content in mushrooms. However, although Gil-Ramirez et al. [52] provided sufficient evidence to prove the first and second points, the third point was challenged by Mohanta [53]. In addition, there is no evidence for the fourth point, only the authors’ speculations. For these reasons, we agree that we should avoid “considering fungi as plants” but the components of fungi should not be ignored. Therefore, without further scientific proof to the contrary, we assume fungi contain flavonoids.

The feed additives used with animals are usually agriculture byproducts or ferments. It was confirmed that fermented products, including waste compost and fungi-fermented products, contain high amounts of flavonoids, which come from the plant-based medium [4]. The phenolic components in fungi play important roles in the antioxidant capacities of animals [51,54,55,56]. It is well known that phenols can chelate free radicals and decrease oxidative stress in animal cells [57,58]. In vivo, phenols enhance antioxidant systems, such as Nrf-2 and glutamate-cysteine ligase catalytic (GCLC) expression, and therefore improve lipid metabolism and meat quality [4,20,59,60].

### 4.3. Ergosterol

First discovered in yeast, ergosterol is commonly found in fungi and has anti-inflammatory effects [5]. Kuo et al. [61] found that ergosterol inhibits the expression of RAW264.7 NF–κB induced by lipopolysaccharides (LPS) and reduces the inflammatory response of TNF-α and other substances (IC50 = 24.5 µg/mL). Ergosterol inhibits the performance of cyclooxygenase-2 (COX-2) but has no effect on nitric oxide production [61]. Not only can ergosterol reduce the inflammatory response in animals, but it is also converted into vitamin D following exposure to ultraviolet light [62]. Additional vitamin D intake increases serum levels in animals [63] and may therefore enhance antioxidant and fat metabolism capabilities.

### 4.4. Adenosine

Adenosine, an end product from the breakdown of ATP, is common in fungi and animal cells [64,65]. It regulates energy utilization in animal cells and reduces the inflammatory response. Adnosine binds to G proteins (A1, A2A, A2B, and A3) to regulate the immune capacity and inhibit the production of harmful enzymes and proteins [64,66]. When combined with different G protein receptors, adenosine improves the growth of macrophages (A1), suppresses the inflammatory response by inhibiting Th1 activities and promoting Th2 activities (A2A), upregulates Th2 activities (A2B), and suppresses immunity (A3) [66]. In *Cordyceps militaris*, there is another special adenosine, 3′-deoxyadenosine, also known as cordycepin [65]. Cordycepin upregulates pro-apoptosis genes such as P53, BCL2-associated X protein, caspase-3, and caspase-9, while downregulating antioxidant gene expression. Apoptosis is triggered by the destruction of mitochondria in the tumor cell, thereby inhibiting the growth of cancer cells in the brain [67,68].

### 4.5. Fungal Cell Wall and Polysaccharides

The fungal cell wall consists mainly of β-glucan, mannoprotein, and chitin [69]. Of these, the first two are very effective at coating LPS and mycotoxins and can reduce their damage [69,70,71]. In addition, β-glucan, mannoprotein, and chitin can regulate immunity, increase antioxidant capacity, and reduce the inflammatory response [70,72]. A common fungal polysaccharide is yeast cell walls.

Yeast cell walls are dominated by mannan-oligosaccharides and β-glucans, with less chitin [70]. Mannan-oligosaccharides and β-glucans reduce the harm of ochratoxin A to broilers and regulate their immunity [70]. β-glucans also reduce the inflammatory response and the harm from LPS [73,74]. The concentration of polysaccharides in *Cordyceps*, as with other functional metabolites, is significantly affected by medium composition (moisture, geographical environment, and climate) [37,75]. In general, *Cordyceps militaris* contains about 3–8% of dry matter and is stored in fruiting bodies and mycelium in solid and liquid media [37,75]. *Cordyceps* polysaccharides enhance immune cell phagocytosis, improve humoral and cell immunity, enhance the activity of macrophages, monocytes, and lymphocytes, and reduce oxidative stress by enhancing the activity of SOD and Gpx. In addition, crude extracts of *Cordyceps militaris* can reduce the amount of lipid oxidation [75].

Polysaccharides derived from *Flammulina velutipes* decrease the pH value in mice intestines and increase both the amount of short-chain fatty acids in mice intestines and serum immunoglobin [34]. In addition to acting as a kind of prebiotic, polysaccharides in edible mushrooms are also well known for their antioxidant and anti-inflammatory capacities [76,77,78,79,80,81]. In mice, the polysaccharides in *L. edodes* and *Phallus atrovolvatus* decrease TNF-α, IL-6, IL-1β, blood urea nitrogen, and uric acid levels in blood serum while increasing antioxidant enzymes such as SOD, CAT, and Gpx in the kidney [77,79]. Accordingly, due to the antioxidant and anti-inflammatory capacities of their polysaccharides, mushrooms have potential as a feed additive which improves animal health.

### 4.6. Enzymes

Fungi produce multiple enzymes during cultivation, which can be divided into three categories: peroxidases, carbohydrases, and phytases. Peroxidases assist fungi in facing the challenge of toxic phenols and increase their environmental competitiveness [80]. Laccase, one of the most well-known peroxidases in mushrooms, degrades lignin in the culture medium [81]. By degrading lignin, fungi effectively use nutrients that are difficult for other microorganisms to consume [82]. Based on this, the fermentation of plant-based feed supplements can reduce lignin content and increase the concentration of hemicellulose [7,8]. Fang et al. [83] further indicated that fungi could transform fiber into volatile fatty acids as carbohydrate enzymes such as laccase efficiently destroyed the structure of lignin.

Once laccase destroys the structure of lignin, cellulase, and hemicellulase further degrade the fiber in plant-based ingredients. As such, fungi reduce the concentration of difficult-to-digest fiber and produce more hemicellulose-based prebiotics in the fermented medium [7,8]. In previous research, cellulose enzyme groups produced by fungi included cellulase, β-glucanase, xylanase, and β-glucosidase [7,84]. In addition to degrading fiber in the culture medium and providing a carbon source for fungal growth, these enzymes increase crude protein and hemicellulose concentrations in plant-based ingredients following fungal fermentation thanks to the destruction of cellulose and condensed nutrients [7,8]. These additional nutrients can be further utilized by animals, suggesting that fungi-fermented plant-based ingredients have potential as feed additives [4,7].

Another common fungal enzyme is phytase. Phytase degrades phytate in plant materials and increases the animal’s use of amino acids and minerals, especially phosphorus [85]. In general, phytase is found in *Aspergillus niger* and *Escherichia coli* [86]. However, Wanzenbock et al. [87] indicated that the phytate content in wheat bran decreased after *P. eryngii* fermentation, which reveals potential phytase activities in *P. eryngii*. Phytase significantly increases the use of feed nutrients by animals and reduces the excretion of inorganic phosphorus [88]. Interestingly, co-fermentation of phytase with fungal microorganisms enhances the efficiency of phytase by increasing the release of phytate from plant-based ingredients [7]. This indirectly reduces the harm to the environment when raising livestock [7,88].

## 5. The Potential Use of Fungal Feed Additives

T.T. Lee’s research team has discussed in detail the effects of fungal feed additives on poultry health. Fungal species investigated include *S. cerevisiae, A. oryzae, A. cinnamomea, Trichoderma pseudokoningii, Cordyceps militaris, Pleurotus ostreatus, Pleurotus eryngii,* and *Aureobasidium pullulans*. Additives were added directly to the diet or a portion of an ingredient was replaced with either pure probiotic powder, the fermented product, or mushroom waste compost. Fungal feed additives may enhance animal production performance; the results are listed in Table 1.

In addition to improving production performance, fungal feed additives could further enhance animal health (Table 2). Both *S. cerevisiae* and *A. oryzae* are ancient probiotics used in the brewing industry. However, they could also enhance broiler health as feed additives. Chuang et al. [7] indicated that the product from co-fermenting 0.1% *S. cerevisiae* or *A. oryzae* with phytase could reduce the inflammatory response in broilers by decreasing the number of *Clostridium perfringens* in the ileum and suppressing inflammation-related mRNA expression, including NF-κB and iNOS. Furthermore, the *A. oryzae* and phytase co-fermented product enhanced villus height in the jejunum. According to the research of Teng et al. [19], villus height and lactic acid concentration in the ileum increased significantly after supplementation with 10% *S. cerevisiae* wheat bran. Previous research revealed that *A. oryzae* contains multiple enzymes, especially cellulase and hemicellulase, which improves the fiber type in feed and enhances nutrient absorption [8,89,90]. A specific kind of yeast, *Aureobasidium pullulans* increases the hemicellulose concentration of soybean hulls and *Pleurotus eryngii* stalk residue by about 1.5 times following fermentation [60]. *Aureobasidium pullulans* fermented soybean hulls and *Pleurotus eryngii* stalk residue could also decrease the ammonia nitrogen concentration in the cecum [14]. Furthermore, Lai et al. [14] indicated that the *Aureobasidium pullulans* ferment contained high amounts of xylanase and mannanase, and these enzymes proportionally increased relative to the days of fermentation.

Among fungal microbes, there is one special species: *Trichoderma pseudokoningii*. Although *T. pseudokoningii* is not a legal feed additive, its high potential in the poultry industry was reported by both Lin et al. [18] and Chu et al. [17]. Supplementation with 10% *T. pseudokoningii* fermented wheat bran enhanced the villus height, thereby increasing the villus height:crypt depth ratio, and the coliform count in the ileum decreased [17]. The addition of 0.1%, 0.2%, or 0.4% *T. pseudokoningii* powder improved animal growth performance in the first 21 days and improved the feed conversion ratio [18]. Furthermore, supplementation with *T. pseudokoningii* powder increased lactate concentration and lactic acid bacteria levels in the cecum [18].

*C. militaris* and *A. cinnamomea* are both well-known traditional Chinese medicines, due to their antioxidative, anti-inflammatory, and antitumor capacities [9,20]. Lee et al. [20,21] reported on the effects of *A. cinnamomea* powder and fermentation (fermented wheat bran) supplements on antioxidant, anti-inflammation, and fat metabolism in broilers. *A. cinnamomea* powder decreased the coliform count and increased lactobacilli in an in vitro test [20]. Supplementation with 0.1%, 0.2%, or 0.4% *A. cinnamomea* powder enhanced SOD and CAT activities in mRNA as well as protein levels in 21- and 35-day-old animals. The expression of inflammation-related mRNA, such as NF-κB and IL-1β, also decreased [20]. Furthermore, the addition of *A. cinnamomea*-fermented wheat bran to the broiler diet enhanced SOD and CAT activities and reduced total cholesterol and low-density lipoprotein levels in broiler serum [21]. Due to its ease of cultivation, *C. militaris* is more popular than *C. sinensis*. However, as with other types of mushrooms, its waste residue is rich in mycelium and other functional compounds, suggesting it has potential as a feed additive. Wang et al. [15] indicated that supplementation with 0.5%, 1%, or 2% *C. militaris* waste residue increased egg mass and eggshell strength and improved the feed conversion rate throughout the entire experimental period. The cholesterol content in egg yolk also decreased after supplementation with 1% and 2 % *C. militaris* waste residue [15]. Furthermore, Hsieh et al. [9] revealed that *C. militaris* waste residue was about 9 times higher in polysaccharide content than *Pleurotus eryngii, Pleurotus sajor-caju*, or *Fammulina velutipes* waste residues. As such, supplementation with *C. militaris* waste residue could significantly increase antioxidant capacity via activated Keap-1 and Nrf-2 mRNA expression [9].

Another common fungal feed additive is mushroom waste compost. As the residue left after cultivation, mushroom waste compost is high in mycelium and wood fiber, which enhances animal antioxidants and adipolysis [4]. Although the real effect of mushroom waste compost on adipose metabolism is unclear, it appears to increase KCTD15 and adiponectin expression, thereby enhancing adipolysis-related mRNA expression [4]. Interestingly, in recent years the anti-obesity capacity of vitamin D, which is converted from ergosterol, has attracted attention [62]. The addition of vitamin D could enhance antioxidant capacity, decrease the total cholesterol and triglyceride levels in serum, and further enhance the peroxisome proliferator-activated receptor γ (PPARγ) and perilipin-2 expression to enhance adipolysis [91,92,93]. Bindhu and Arunave [94] also indicated that *Pleurotus ostreatus* and its bioactive anthraquinone could enhance the PPARγ and CCAAT enhancer-binding protein α (CEBPα), thereby decreasing adipose accumulation. This suggests that the anti-obesity capacity of mushrooms may be due to ergosterol and anthraquinone intake.

Several papers point out that mushroom waste compost could enhance antioxidant capacity [4,6,16]. By enhancing Nrf-2 and GCLC mRNA expression, mushroom waste compost could upregulate the antioxidant capacity of poultry and further decrease MDA concentration [4]. Based on its high fiber content, the gut barrier would also be improved by supplementation with mushroom waste compost [4]. Interestingly, Wang et al. [6] indicated that a 10% supplementation of wheat bran fermented by the remaining mycelium in *P. eryngii* mushroom compost could also enhance CAT activities. Overall, whether they’re used in traditional Chinese medicine or are common edible species, fungi play an important role in upregulating animal health and could protect against stress. Based on the effects of the different fungal feed additives on growth performance and animal health, supplementation with 0.1% *A. cinnamomea,* 0.5% mushroom waste compost, or 2% *C. militaris* waste residue are recommended [4,15,20]. Furthermore, in addition to avoiding illegal or harmful fungi, it is not recommended to add more than 5% of any fungal feed additive since better results were achieved with a small amount (<2%) (Table 1).

## 6. Future Perspectives

In general, probiotic discussions have focused on bacterial microorganisms and the potential use of fungi has long been ignored. Through this review, we have tried our best to fully explain the potential uses of fungi. Fungi can enhance antioxidant capacity and fat metabolism in animals and maintain intestinal health. However, pathogenic fungi should be avoided, even if probiotic fungi can offset their negative effects. The most suitable amount depends on the species and type of fungi. Overall, the value of fungi as probiotics or functional feed additives has been highlighted in this review.

## Figures and Tables

**Table 1:** The effects of fungal feed additives on animal growth ^1^.

Animal Type	Treatment	Rearing Period	Body (Egg) Weight	Feed Conversion Rate	References
Broilers	0.5% *A. pullulans* fermentation ^2^	22–35 d	+12% *	−4%	[14]
		1–35 d	+8% *	−3%	
Hendrix laying hens	2% *C. militaris* wastes residue	5–8 weeks	+4% *	−10% *	[15]
		9–12 weeks	+3% *	−5% *	
		0–12 weeks	+7% *	−6% *	
White Roman geese	5% mushroom waste compost	5–8 weeks	+2%	0%	[16]
		9–12 weeks	−1%	+31%	
Male broilers	0.1% *T. pseudokoningii* powder	1–21 d	+10% *	−4%	[18]
		22–35 d	+6%	−3%	
Broilers	10% *P. eryngii* mushroom compost fermented wheat bran	1–21 d	+2%	−8%	[6]
		22–35 d	+3%	0%	
		1–35 d	+2%	−3%	
Male broilers	0.1% *A. cinnamomea*	1–21 d	+14% *	−1%	[20]
		22–35 d	+10% *	−5%	
		1–35 d	+11% *	−7%	
Male broilers	10% *A. cinnamomea* fermented wheat bran	1–21 d	+7% *	−5% *	[21]
		22–35 d	−1%	+1%	
		1–35 d	−2%	+2%	
Male broilers	0.5% mushroom waste compost	1–21 d	+3%	−6% *	[4]
		22–35 d	+5%	+5%	
		1–35 d	+4%	−7%	

^1^ All percentage changes calculated by the difference between the treatment group and control group. ^2^ 75% *Aureobasidium pullulans* fermented soybean hulls in combination with 25% *Pleurotus eryngii* stalk residue * Significant difference between control and treatment group (*p* < 0.05).

**Table 2:** The effects of fungal feed additives on animal health.

Animal Type	Treatment	Functional Components	Functions	References
Broilers	0.5% *A. pullulans* ferment ^1^	- ^2^	Increased SOD and CAT activities, ileum villus height and lactic acid bacteria number in cecum	[14]
Hendrix laying hens	2% *C. militaris* waste residue	Cordycepin, cordycepic acid, crude polysaccharides, flavonoid, adenosine, and crude triterpenoid	Increased egg mass and eggshell strength; improved feed conversion rate throughout the entire experimental period; decreased cholesterol content in egg yolk	[15]
White Roman geese	5% mushroom waste compost	-	Increased SOD activities and decreased MDA content in serum; improved flavor, color, and acceptability on sensory evaluation	[16]
Male broilers	10% *T. pseudokoningii* fermented wheat bran	Cellulase, xylanase, and reducing sugar	Decreased coliform count and increased villus height in ileum	[17]
Male broilers	0.1% *T. pseudokoningii* powder	Phenols, xylanase, and cellulase	Increased SOD and CAT activities, jejunum villus height, and lactic acid bacteria levels in cecum	[18]
Broilers	10% *S. cerevisiae* fermented wheat bran	-	Increased villus height and lactic acid content in ileum	[19]
Male broilers	0.4% *A. cinnamomea* addition	Crude triterpenoids, crude polysaccharides, and phenols	Enhanced Nrf-2, GCLC, SOD, CAT and HO-1 mRNA expression and decreased NF-κB and IL-1β mRNA expression	[20]
Male broilers	10% *A. cinnamomea* fermented wheat bran	Crude triterpenoids, crude polysaccharides, and phenols	Enhanced SOD and CAT activities and decreased total cholesterol and low-density lipoprotein content in serum	[21]
Male broilers	0.1% *S. cerevisiae* or *A. oryzae powder*	Xylanase, protease, cellulase, and ß-glucanase	Decreased the number of *Clostridium perfringens* in ileum and suppressed inflammation-related mRNA expression	[7]
Male broilers	0.5% mushroom waste compost	Crude triterpenes, phenols, flavonoids, gallocatechin, and epigallocatechin	Increased antioxidant capacity, adipolysis, and gut barrier expression	[4]
Female broilers	1% *C. militaris* waste residue	Polysaccharides, triterpenes, phenols, and flavonoids	Enhanced antioxidant-related mRNA expression	[9]

^1^ 75% *Aureobasidium pullulans* fermented soybean hulls in combination with 25% *Pleurotus eryngii* stalk residue; ^2^ The symbol “-” indicates the author did not mention the functional components.

## Abbreviations

TNF-α	Tumor necrosis factor alpha
Gpx	Glutathione peroxidase
SOD	Superoxidase dismutase
MDA	Malondialdehyde
TLR4	Toll-like receptor 4
MyD88	Myeloid differentiation primary response 88
COX-2	Cyclooxygenase-2
CAT	Catalase
NFκB	Nuclear factor kappa B p 65
iNOS	Inducible nitric oxide synthases
IFN-γ	Interferon-γ
IL-1ß	Interleukin-1ß
Nrf-2	Nuclear factor erythroid 2–related factor 2
GCLC	Glutamate-cysteine ligase catalytic
KCTD-15	Potassium channel tetramerization domain-containing 15
CEBPα	CCAAT-enhancer-binding proteins-alpha
CPT-1	Carnitine palmitoyltransferase I
PPAR-γ	Peroxisome proliferator-activated receptor gamma
